# Sex/gender entanglement: A problem of knots and buckets

**DOI:** 10.1186/s13293-025-00758-9

**Published:** 2025-10-30

**Authors:** Donna L. Maney, Annie Duchesne, Giordana Grossi

**Affiliations:** 1 Department of Psychology, Emory University, Atlanta, GA 30322 USA; 2 Department of Psychology, University of Northern British Columbia, Prince George, BC Canada; 3 Department of Psychology, State University of New York at New Paltz, New Paltz, NY USA

**Keywords:** Entanglement, Gender/sex, Sex/gender, Sex contextualism, Operationalization

## Abstract

When used as variables in biomedical research, sex and gender can be difficult to operationalize and measure. Questions have arisen about whether either category is stable or causally meaningful in a research context. Here, we discuss some of the limitations of using both or even one of these categories in correlational or experimental work. We argue that attempting to draw a distinction between sex and gender can reignite the nature/nurture debate, inadvertently bringing outdated metaphors and assumptions about innateness and causation into our research. Many researchers, including ourselves, have described sex and gender as separate collections of causal factors (which we describe as a “bucket” metaphor) or as entangled (a “knot” metaphor). Because they regard sex and gender as conceptually separable and internally consistent, such metaphors have limited value for understanding the drivers of diversity in our data. Rather than continuing to reify sex and gender as distinct buckets or threads of explanatory variables, we call for deconstruction of these categories by focusing instead on clearly operationalized, instantiating variables that researchers can manipulate or measure. Our proposed approach differs from recent, similar calls in that we are not suggesting the exclusion of a sex/gender category from statistical models; instead, we recommend keeping it—not as a representation of biological reality, but as a tool used under a careful set of assumptions. We provide example datasets to illustrate how a sex/gender category can, when thoughtfully operationalized, be used to improve statistical rigor and inferential precision. In addition, we advocate for attention to variation within sex/gender, which is more informative in investigations of mechanism than comparing means across sex/gender categories.

**Supplementary Information:**

The online version contains supplementary material available at 10.1186/s13293-025-00758-9.

## Highlights

Recent calls to consider gender, as well as sex, in biological and biomedical research come from an appreciation of the need to acknowledge complexity. Here, we consider the utility of using sex or gender categories to represent that complexity.

The concepts of sex and gender, despite originating from very different disciplines and philosophies, are now regarded oppositionally in terms of the nature-nurture dichotomy, a framework that has been rejected in the biological and developmental sciences.

Other authors have recently advocated for moving past binary sex and gender categories to instead consider instantiating, measurable variables of interest. We discuss the pros and cons of this approach, and, using sample datasets, present alternatives that can address its shortcomings.

We advocate keeping a single, general sex/gender category—not as a representation of biological reality – but instead as a tool used to improve analytical rigor and inferential precision.

**Supplementary Information:**

The online version contains supplementary material available at 10.1186/s13293-025-00758-9.

Recent calls to consider gender, in addition to sex, in biomedical research reflect the need to better represent complexity and diversity in human phenomena. The addition of “gender” as a (usually categorical) variable to study designs acknowledges that “sex” cannot capture factors such as gender identity and gendered life experiences, which are essential to understanding human biology and development. Guidelines on how to incorporate sex, gender, or both into research designs typically draw sharp distinctions between the two concepts. Sex is typically described as “biological” whereas gender usually refers to the “cultural,” “social,” or “behavioral” [1]. Attempts to assess their independent predictive values have suggested that the two types of categorizations can, in some cases, lead to different conclusions about differences between women and men (e.g., [2–4]).

A stated goal of this special issue of *Biology of Sex Differences* is to move away from thinking of sex and gender as dichotomized and separable, and to ask instead how “biology” and “culture” interact and move through each other [5]. Indeed, the conceptualization of sex and gender as “entangled” [6] is making its way more and more into the biosciences [7]. DuBois et al. [8] argued that “entanglement recognizes that sex and gender are literally inseparable and co-constituted.” They offered as an example the hormone testosterone, a factor that is often considered part of the definition of sex [9] but is nonetheless sensitive to social experiences. But even this language embodies two sides—two distinct entities: the hormone and the social experience. The term “entanglement” conjures a knot that, although messy, has two identifiable threads. Thus, this conceptualization requires us to articulate and understand the *boundaries between* sex and gender; between “biology” and “culture” [10, 11]. These boundaries are not as clear, informative, or necessary as we might think. Here, we first consider whether classifying variables into two separate categories, sex and gender, is serving us well in our attempts to understand human biology. Second, we consider whether we even need one category.

## The fuzzy boundary between sex and gender

The distinction between sex and gender has been a focus of a massive education campaign targeting biomedical researchers who use the terms interchangeably. Intended to raise awareness about the meanings of the terms, guidelines have appeared in biomedical journals (e.g., [12–14]) and trainings are offered on the web sites of funding agencies (see [1]). Some of these materials, however, are themselves confusing and inconsistent, highlighting conceptual problems with how ‘effects’ of sex and gender are being attributed. In the online short course on sex and gender at the Canadian Institutes of Health Research [15], the distinction starts out simple enough: “sex” is said to be “a set of biological attributes” whereas “gender” is “socially constructed roles, behaviors, expressions and identities.” In the subsequent quiz, the reader is asked “which of the following is a gender-related variable?” The answer choice “Ebola virus transmission” is deemed correct, because it is related to the *behavioral* role of women as caregivers. The answer choices related to hemoglobin levels, ferritin levels, cardiac ejection fraction, heart failure, and kidney function are deemed incorrect. According to the explanation, these variables “are sex-related” because they are “physiological,” ruling out an influence of gender in their function or development.

Of course, it is possible to identify many ways that blood, the heart, the kidneys, or any other tissue might be affected by gendered exposures – the same training course points out gendered exposure to formaldehyde in nail polish, for example, as potentially affecting physiology. The tendency to attribute all physiological differences to sex over gender mirrors the most common ways that biomedical researchers interpret sex differences in their data – if the outcome measure is something physiological, particularly in the brain, a difference is typically assumed to be caused by “biological” factors, most commonly gonadal hormones [16]. This assumption carries important risks, such as misdiagnosis and misattribution in clinical settings [17, 18], pathologization of being female (e.g., [10, 19, 20], and exclusion of trans identities (e.g., [21]). Clearly, there is confusion and disagreement about boundaries between sex and gender and how to draw them. Instead of attempting to categorize variables as one or the other, it would be more helpful to re-evaluate the concepts of sex and gender themselves and assess their value as separate entities.

## Sex vs. gender: A nature/nurture redux

The concepts of sex and gender emerged from very different disciplines and scholarly traditions. The term “sex” has historically been used in the biological sciences whereas our current understanding of “gender” is grounded in queer, feminist, and gender studies [22–24]. The practice of pitting them against each other, as complements or opposites, emerged in the fields of psychology and anthropology during the mid-twentieth century to meet a perceived need for distinct terms ([25–27], see also [28]). Indeed, distinctions between the ‘biology’ and the ‘social construction’ of women and men were central to the work of early feminist scholars such as de Beauvior ([29] see also [30]). In the case of gender *identity*, which in many individuals is distinct from sex assigned at birth, a separate term is strongly warranted. The need for this term does not, however, deny “biological” contributors to, or effects of, gender identity. Gender identity is part of a person’s biology and can profoundly affect physiological systems. The deep situatedness of all variables in dynamic and complex systems illustrates the difficulty of assigning kidney function or Ebola transmission to a “biology” bucket or a “culture” bucket. Most variables cannot be sorted as such, and we argue here that attempting to do so may not prove fruitful to advance our understanding of the roles of sex and gender in human biology and health.

As commonly conceptualized in the biomedical sciences today, sex and gender represent a rebranding of *nature* and *nurture*—a false dichotomy that has vexed and beleaguered scientists and philosophers for decades, if not centuries. Although the conceptual parsing of native dispositions and environmental influences appeared even as early as the sixteenth and seventeenth centuries (reviewed by Keller [11]), the first to explicitly pit nature and nurture against each other in so many words was the eugenicist and statistician Galton [31]. This dichotomous way of thinking about development was later co-opted by Lorenz (e.g., [32]), who argued that whereas some behaviors are “innate,” relying simply on maturation for their development, others are “learned” and require input from the environment. Although the innate-learned dichotomy left a profound mark, not only on the study of animal behavior but on other fields such as psychology [33] and behavioral genetics [34], it did not go uncriticized. For example, Lehrman [35] questioned “whether experiments based on the assumption of an absolute dichotomy between maturation and learning ever really tell us *what* is maturing, or how it is maturing?” (p. 344). If learning can somehow be ruled out as a factor shaping a behavior, he argued, how do genes alone cause that behavior to develop? Continuous *interaction* is necessary—not between the environment and genes, but between the environment and the individual.

Lehrman’s critique laid the groundwork for an alternative way of thinking, eventually dubbed *interactionism,* that was embraced by many as the demise of the nature-nurture debate (reviewed by [36–39]). But, even as the old dichotomies (nature-nurture, biological-social, inside-outside, innate-learned) began to be routinely rejected [40], the interactionist viewpoints continued to reify and privilege aspects either inside (e.g., genes, hormones) or outside (e.g., society, the environment) the individual. As Oyama [38] argued, besides not being supported by empirical evidence, a major problem with such frameworks is that they assume some form of preformationism: the “information” for building an organism preexists its development, and the process that moves it from one state to another is always somewhere else, in an infinite regress. Interactionist views do not solve the problem of preformationism; they simply combine two sets of causes without actually engaging with their interaction.

Even decades after Lehrman’s influential paper, although psychology and biology textbooks of the time declared the nature-nurture issue dead, dichotomous thinking about genes vs. learning was alive and well (e.g., [37, 38]). Authors at that time would eschew the learned-innate dichotomy in one paragraph, then in the next, use slightly altered language with essentially the same meaning. We have often seen the same phenomenon in discussions of sex and gender, that is, the dismantling of the nature-nurture dichotomy in one paragraph, followed by its linguistic reconstruction in the next. Even the most recent conceptualizations of entanglement conjure metaphors of looping relationships between sex and gender—entangled knots and the like (see [7, 8]). The metaphors may have become more complex over time, but the underlying conceptualization of a dichotomy has persisted. Continuing to contemplate sex and gender as interacting and entangled will not enable us to escape from an outdated nature/nurture framework, even as we attempt to move past it [10, 11].

The quiz question from the CIHR training module clearly illustrates the problems with attempting to assign any variable to a “sex” or a “gender” bucket. Sex- and gender-related variables ultimately melt together, and understanding one variable from a particular bucket does not necessarily inform understandings of other variables in that bucket. That is, discovering a relationship between nail polish and a protein expressed in the kidneys will not help us understand the relationship between a gendered eating disorder and a protein expressed in the intestines. The nail polish, the kidney protein, the eating disorder, and the intestinal protein are four variables with complex relationships – knowing which bucket each one belongs to is unlikely to provide significant insight into those relationships. Sex and gender are each, as buckets, too large and heterogeneous. The constructs themselves do not meaningfully narrow down the causes of variation. Instead, they simply delineate assumptions about nature and nurture, inside and outside, as alternatives.

## Undoing sex and gender

After questioning whether sex and gender can be empirically separated as categorical variables, we must now consider whether either category is necessary for the scientific pursuit of causal, mechanistic explanations. In the biological and biomedical sciences, although sex and gender are often treated as causal variables, they are in fact classification systems [41]. The National Institutes of Health and the National Academies of Sciences, Engineering, and Medicine [9]) define sex as “a multidimensional construct based on a cluster of anatomical and physiological traits that include external genitalia, secondary sex characteristics, gonads, chromosomes, and hormones.” How can a researcher best use such a definition to assign participants or animals to a sex category? Opinions vary regarding which single trait can or should define a body as male or female; some have argued that none of them can [42]. The traits used to assign participants, animals, or samples to sex categories can vary by field, by lab, or even from study to study within the same lab [41]. But more importantly, the chosen sex-definitive trait (typically external genitalia at or shortly after birth) is almost never suspected as a *cause* of sex-related variation. If a statistical difference is found between females and males, we point our fingers at *other* parts of the sex construct, for example ovarian hormones or gene expression, to hypothesize about causality [16, 43, 44]. Thus, there is a profound and interesting mismatch between what we say sex *is* and what we say sex *does*.

As a variable in a statistical model, gender is similarly problematic. Like sex, it is defined as a multidimensional construct [9]. But its dimensions and components vary from individual to individual and depend on context, presenting extraordinary challenges to standardized operationalization. Several available instruments, for example the Gender-related Variables for Health Research (GVHR), consist of questionnaires that collapse many gender-related metrics into more manageable factors or scores ([45]; see also [46]). Such measures can provide excellent options for *controlling* for gender-related variation in data but are not as useful for exploring factors that may *predict* or *cause* that variation because they can flatten and mask variables that could be explanatory [47].

Sex and gender categories fail as empirical variables because they do not represent tangible, measurable traits that can influence an outcome measure. This failing was noted even by Unger [26], one of the first scholars to call for a distinction between them. Today, other authors argue for deconstructing these fraught categories, moving instead to instantiating, measurable variables that *can* explain variation [16, 41, 43, 48–53]. A potential interpretation of these authors’ recommendations is that the categories of sex and gender (hereafter referred to collectively as s/g) might be completely removed from statistical models and replaced by s/g-related variable(s) that could be causal for s/g-related differences observed in the data. For example, researchers interested in variation in spatial learning could include a s/g-related variable of interest, such as plasma estradiol, in their models instead of s/g category itself. Moving past s/g categories in this way solves the bucket problem because we do not need to assign variables of interest to either bucket. We simply measure what we are interested in, test for relationships, and draw conclusions that relate precisely to those variables. Whether the variables count as “sex” or “gender” is irrelevant in this context because our focus is instead on potential predictive and/or causal mechanisms that will move our science forward without the sex and gender buckets and the biases that come with them.

## Moving beyond binary categories while maintaining inferential precision

Approaches that move beyond s/g categories, replacing them with one or more tangible, s/g-related variables, bring focus and precision to the research question; however, a significant oversight of such approaches is that they do not account for complex arrays of covarying s/g variables that could also explain observed associations. In the example above, an association between learning and estradiol does not provide strong evidence of a direct relationship because estradiol is likely confounded with countless other s/g-related variables that could create the illusion of an informative association but are not in the model. An association between spatial learning and estradiol could be driven, for example, by any number of gendered experiences such as toys, sports, or professions—because these experiences are correlated with gender, they are also likely correlated with plasma estradiol regardless of any actual connection. The same applies even in non-human animal models, where traits associated with plasma estradiol cannot be attributed directly to it or any other single variable of interest. Any association between a single s/g related variable and any outcome measure is confounded by practically every other aspect of what makes up sex and gender. No strong conclusion can be drawn about any s/g-related variable of interest without somehow controlling for this confounding co-variation.

**Fig. 1:**
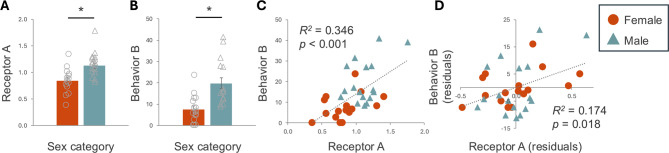
A hypothetical example illustrating exploration of sex-related variables. Imagine we have found a statistically significant sex difference in the density of receptor A in mice (**A**), and we are interested in whether it is involved in behavior B, the frequency of which also differs by sex category (**B**). If we were to test for a relationship between the two variables A and B using an approach that eschews sex category, we would find a statistically significant correlation but it would be logically explained simply by sex differences in the two variables (**C**); in Panel C, most of the females are on the low end of both the receptor and the behavior, and the males on the high end. This distribution of data does not demonstrate convincingly that the two variables are related independently of sex, because there are many other sex-related variables that could explain the association—that is, they affect both A and B such that a potentially spurious association emerges. If we want to explore the possibility of a more direct relationship between the receptor and the behavior, we could test for a partial correlation, holding sex category constant (**D**). This approach removes the variability due to sex category from both A and B, thus accounting for a large amount of variation that does not interest us. Panel D shows the association between A and B after the variability attributable to sex category has been removed from each (the “residuals”); a statistically significant partial correlation, controlling for sex category, tells us that the relationship between A and B cannot be explained solely by their respective relationships with sex category. *p < 0.05. All analyses were conducted in SPSS v. 31. Data used to make the figure are available in Supplementary Table 1.

This conundrum highlights why s/g category, despite its imprecision, is a popular addition to statistical models. It single-handedly accounts for innumerable factors, known and unknown, measurable and immeasurable, biological, environmental and cultural, that are all associated with each other, often inextricably. The practice of “controlling for” s/g category, that is, including it in a statistical model as a covariate, has been criticized, however, because it removes variation explained by s/g instead of treating s/g as a variable of interest [54]. Thus, we have two problems: first, that s/g category is an ill-defined variable that masks important within-group variation; and second, adding it to a statistical model can mask potentially important effects and relationships. Both problems might be addressed by incorporating *two* variables: a precise s/g-related variable of interest and a carefully operationalized s/g category as the background we control for. In Fig. 1, we use a hypothetical dataset to illustrate how s/g category could be used to control for s/g-related confounds while considering a potentially explanatory s/g-related variable. In the example, which shows a potential relationship between a hormone receptor and a behavior, controlling for s/g category allows us to account for potentially confounding s/g related variability, increasing the predictive power of the variable of interest. This type of approach can provide valuable information about potential mechanisms underlying s/g differences, which will ultimately inform medical practice far more than simply comparing between two s/g categories [41].

There are two caveats to this approach. First, since by definition both variables in the model are related to s/g, there could be little residual (unique) covariation between them once s/g is controlled. This “multicollinearity” could obscure a relationship between the receptor and the behavior even if they are related independently of s/g. The second caveat is that testing for association provides only weak evidence of causation. Experimentally manipulating the expression of receptor Y would be an excellent next step to test for causality, but such approaches are often not available particularly when working with human participants.

The approach illustrated in Fig. 1 will be valuable only when both the sex-related variable and the more general s/g category are carefully operationalized. Operationalization of s/g is typically missing from research in the biosciences, however; authors rarely indicate how they assigned animals, participants, or samples to s/g category. The method of assignment, for example anogenital distance for rodents or self-report for human participants, is reported less than 1% of the time (unpublished data, see Supplementary Methods), which contributes to its nebulousness. When it is appropriate to control for s/g variation by including it in a statistical model, it is but a simple step to explain its operationalization in the published article. Including this information takes little time or space and will help normalize clear, explicit operationalization of all variables.

## Trait-makers vs. difference-makers

Figure 1 brings to mind a problem germane to the nature-nurture debate since its inception. As pointed out by many authors over the last century, including Fisher [55], Lewontin [56], and Keller [11], considering the causes of a particular trait often conflates two very different questions. First, what causes the trait itself – how do distinct causal factors contribute to that trait and how do we parse those factors? Second, what explains *differences* in the trait between two populations—is that difference explained by a difference in a causal factor? These two questions must be asked separately, using different approaches. Some have argued that the first question, which pertains to development, is difficult to answer in day-to-day scientific practice (see [11]).

The second question can be addressed statistically, and in fact lies at the heart of modern analytical approaches to data [11, 55, 57]. In the example in Fig. 1, we are not particularly interested in the sex differences in the behavior or in the receptor, apart from how they might be related to each other. For example, higher frequencies of the behavior in males might be explained simply because males have more of the receptor—that is, there might be nothing about being male, other than the receptor density, that affects the relationship between the receptor and the behavior. Showing a sex difference in both measures (Fig. 1 A, B) serves as crude evidence that they *might* be related; this hypothesis is tested directly in the partial correlation (Fig. 1D), which provides stronger evidence of that relationship independently of sex—that is, the relationship between the two variables might be the *same* in females and males. Of course we have more to do before establishing a causal relationship. But even causal evidence addresses the cause of the *difference*, not the cause of the *trait*.

In real life, relationships are not so simple. Keller [11] argued that the above approach to explaining differences relies on a huge assumption: non-interaction between the two measures. Complex interactions among s/g-related variables often muddy the direct relationships. For example, in our mice in Fig. 1, the binding of the receptor to its ligand may depend on yet another sex-related variable that we have not measured. Performance of the behavior itself may depend on a sex-related morphological feature (e.g., size, external genitalia). In our nail polish example, men who wear it may be more likely to engage in kidney-affecting behaviors than women who wear it. That is, the pathway from trait A to trait B may itself depend on other s/g-related influences [13]. Therefore, we need to know whether individuals of one s/g category are more sensitive or susceptible to the influence of a factor than individuals of another s/g category, such that the factor has a larger effect in one s/g than another.

One common approach to understanding such interrelationships is to test for effects that are “specific” to one s/g category. Such effects are often said to be “sex-specific,” “gender-dependent,” or to occur in “males but not females” and so on. For example, when testing the effect of a treatment or intervention, we might disaggregate our data by sex category and compare that effect between females and males. This practice can, in theory, reveal complex relationships between s/g and our variables of interest, helping to identify potential s/g-related mechanisms for future investigation.

**Fig. 2:**
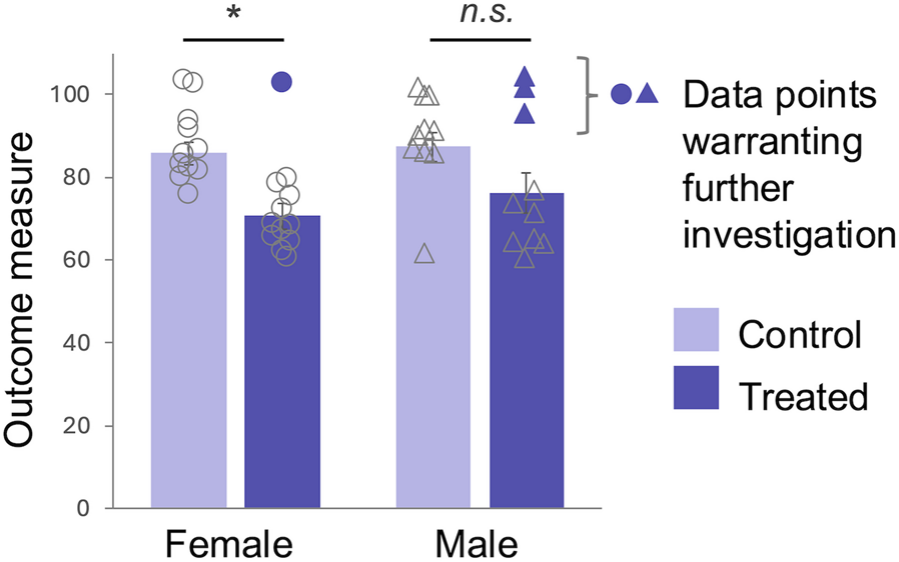
Informative variability masquerading as a sex difference. These data are loosely based on a recent published article on mice, in which the authors concluded that a treatment affected the outcome measure only in females. In the article, however, the sexes were never statistically compared. When they are compared by testing for a sex-by-treatment interaction in an analysis of variance, the difference between the responses in females and males is not statistically significant (p = 0.583). Thus, we do not have evidence that the males and females responded differently to the treatment (Rich-Edwards and Maney, [61]). The inability to detect a statistically significant decrease in the males is due to three male non-responders (noted on the graph in dark purple); the rest of the males responded like most of the females. The four non-responders, one of which was female, represent an opportunity for further exploration of the mechanisms underlying differential responses, which according to these results, did not depend on sex. *p < 0.05 based on inappropriate within-sex tests; n.s., non-significant. Data used to make the figure are available in Supplementary Table 2

Because this approach collapses often tremendous variation into broad s/g categories, however, it tends to mask, rather than reveal, interesting variability that would advance understandings of mechanism. This issue is particularly worrisome when means are compared [58, 59]. Figure 2 shows an example of a research design in which the use of sex categories causes the results to be misrepresented and potentially important findings to be missed. Here, if researchers detected a statistically significant effect of a treatment in female but not male mice; they would then likely conclude that the sexes responded differently to the treatment (see 60). But a quick look at the data shows that the females and the males responded similarly to the treatment. In the example, the failure to detect a statistically significant effect in the males is likely due to interesting variability, which happened to be higher in the treated males. A different strategy, involving direct statistical comparison across sex category (i.e., testing for a sex by treatment interaction) and attention to the outliers, would prevent the spurious finding of a sex difference and allow attention to be focused on the most informative data.

Both examples (Figs. 1 and 2) call attention to the fact that within-group variability is often more interesting and potentially informative than variability between groups. In the first example, s/g category is used first to identify a candidate mechanism, then to control for confounding s/g-related variables while exploring that candidate. In the second example, the inappropriate analytical treatment of s/g category has hindered the investigation by distracting from the data that point toward potential mechanisms. In both examples, it is the within-category variation, not the category itself, that provides the information relevant to identifying causal factors.

Binary, categorical variables have other costs. For example, their use requires assignment of all animals or participants to one category or the other. This issue is one of the rationales for “sex contextualist” approaches in which one or more precisely operationalized, sex-related variables are used in place of sex category as a variable of interest [41, 43, 48–51, 53]. Precise operationalization of s/g category can ameliorate, but not solve, this problem. Ultimately, if s/g category is included to control for a multitude of unknown nuisance variables in the data, it is less important to strive for homogeneity within the category. In fact, diversity within the category is essential for it to do its job. In all cases, the criteria for assigning participants to groups should be carefully chosen to reflect the context and goals of the study [41, 49].

Truly moving beyond s/g categories cannot be done by comparing averages. Simply comparing means, as one does in a t-test or an analysis of variance, does not provide a clear picture of empirical distributions. Particularly in research that considers s/g, as noted above, comparing averages brings a number of limitations that can lead not only to misinterpretation of the data, but that hinder the discovery of variability that would contribute to the goal of precision medicine [41, 48, 52, 58, 59, 62]. Splitting the population into binary groups for the purposes of delivering health care brings negligible benefits to precision but carries large costs by reinforcing and magnifying perceptions of difference [19, 58, 63, 64]. Instead, investigating variation related to carefully operationalized, continuous variables will better inform the development of customizable treatment options [41, 58, 65].

## Conclusion

Nearly five decades ago, groundbreaking feminist psychologist Unger [26] wrote, “The questions of sex differences are someone else's questions—They do not, of themselves, illuminate the mechanisms that create such differences. In fact, they may obscure the origin of such differences by leading us to believe that biological explanations are sufficient for understanding [behavior]” (p. 1089). Here, we argue that just as sex differences do not illuminate the mechanisms that create difference, neither do gender differences. Adding a gender bucket as a source of potential mechanisms obscures the origins of difference even further by introducing the distraction of a nature-nurture dichotomy. Gender diversity *itself* has many causes and effects, few of which can be assigned to an exclusively “biological” or a “cultural” category. The categories of male/female, man/woman are broad and extremely heterogeneous. Considering the sources of that variation as somehow belonging to a sex or gender bucket sacrifices precision and expends resources that could be used to ask more informative questions.

No matter the model organism, it should not be necessary to categorize influences or traits according to inside/outside, genes/environment, sex/gender, nature/nurture. Rigorous scientific approaches do not require such disentanglement. We can use the knot, in its entangled state, as a tool to discover the causal mechanisms explaining variability. The trick is to recognize what the knot itself represents: an amalgam of mostly unknown and potentially immeasurable nuisance variables that make up a rich background against which we can bring our s/g-related variables of interest into focus. Only by leaving s/g entangled can we leverage it to explore those factors and understand how they contribute to human diversity.

## Supplementary Information

Additional file 1

Additional file 2

## Data Availability

All data generated or analyzed during this study are included in this published article and its supplementary information files.
