# Supplementary material for: Sex/gender entanglement: A problem of knots and buckets
Source: Biol Sex Differ. 2025 Oct 30;16:85. doi: 10.1186/s13293-025-00758-9 (PMC12577278; doi:10.1186/s13293-025-00758-9)
Supplement: Supplementary file 2 — Additional file 2 [file 13293_2025_758_MOESM2_ESM.pdf]

Maney, D. L., Duchesne, A., and Grossi, G. (2025). Sex/gender entanglement: A problem of knots and buckets. *Biology of Sex Differences*.

Supplementary Methods

Calculating the proportion of articles in which s/g is operationalized

A sample of 1300 articles was drawn from 86,000 articles published in 2023 in 66 research areas (e.g., Endocrinology, Neurosciences, General Medical Sciences, etc.) defined by Web of Science. The articles were manually coded by four undergraduate students and one graduate student using SysRev online software. Of the 1300 articles, 379 were excluded because they were not research reports, not in English, they were non-biomedical (e.g., veterinary, ecological), or the studies were not conducted on vertebrates. Of the remaining articles, 66 were further excluded because s/g was not specified. The remaining 855 papers were coded regarding whether s/g was operationalized—that is, whether the authors explained criteria used to assign animals or participants to s/g category. For a non-human animal study, a statement such as, "sex was determined by anogenital distance" would be scored as operationalization. For a human study, a statement such as "participants identified their gender by self-report during face-to-face interviews" or "sex was obtained from medical records" would be scored as operationalization.

Sex or gender was operationalized in only six of the 855 papers (0.7%). All six papers focused exclusively on humans. Sex or gender was determined by self-report (n = 2) from medical records (n=2), or using information in a database (n=2). Sex was not operationalized in any studies conducted on non-human animals.
